# Design and evaluation of a systematic finger-based intervention for early numeracy in 5- to 6-year-olds

**DOI:** 10.1038/s41598-026-43286-1

**Published:** 2026-03-26

**Authors:** Stephanie Roesch, Melissa Conze, Korbinian Moeller

**Affiliations:** 1 Hector Research Institute of Education Sciences and Psychology, University of Tübingen, Tübingen, Germany; 2 Department of Psychology, University of Tübingen, Tübingen, Germany; 3 Department of Mathematics Education, Loughborough University, Loughborough, UK; 4 LEAD Graduate School and Research Network, University of Tübingen, Tübingen, Germany; 5 Institute of Psychology, UMIT-Tirol Private University of Health Science and Technology, Hall in Tirol, Austria

**Keywords:** Early numeracy, Finger counting, Embodiment, Intervention, Psychology, Human behaviour

## Abstract

Early numeracy is a building block for later mathematics achievement in school. Recently, the use of fingers in early numeracy instruction received increasing attention as a prominent example of embodied cognition. Accordingly, the present study developed and evaluated a finger-based intervention for early numeracy skills (involving 12 sessions of 30 min each) that (1) systematically aligns with early numeracy development on all three levels (counting, cardinality understanding, and basic arithmetic) proposed by a recent model of numerical development and (2) using fingers as primary and embodied manipulatives. In a pre-posttest intervention design, 33 5-to-6-year-old children received the finger-based numeracy intervention and were compared to children of a business-as-usual control group (*n* = 37). Results indicated a significant medium-sized beneficial effect on early numeracy, whereas no significant improvements were observed for spatial working memory or fluid reasoning. Interestingly, the number of finger users increased from pre- to posttest but did not differ between children who received or did not receive the finger-based intervention. Irrespective of group assignment, finger users consistently outperformed non-finger-users in early numeracy. Taken together, these findings highlight that finger use is a powerful and beneficial approach for fostering the development of early numeracy.

**Supplementary Information:**

The online version contains supplementary material available at 10.1038/s41598-026-43286-1.

## Introduction

Early numeracy such as counting, magnitude understanding, and basic arithmetic is widely recognized as important building blocks, not only for later mathematics achievement in school^1–4^ but also for more general live prospects^5^. These findings underscore the relevance of fostering numeracy during early childhood using well-designed and effective interventions^6^. To be effective, such interventions should closely align with and facilitate typical developmental progression as reflected by models of early numeracy development and specified learning trajectories^7,8^. By adhering to these frameworks, interventions ensure that activities involved are within the developmental capacities of young children. Additionally, they systematically build on and expand children’s prior knowledge and strategies following the idea of a hierarchical development of numeracy skills and concepts.

In recent years, finger use in early numeracy instruction has received increased attention. In particular, using fingers for counting and initial calculations was argued to be a key example of embodied cognition^9,10^, which assumes influences of physical actions (like finger use) on cognitive processes (in this case the processing of numerical information). In this context, fingers are seen as a powerful multisensory “tool” to learn about numeracy concepts such as counting, cardinality^11^, and part-whole relations^12^. For instance, when four fingers are extended while saying “this is four”, perception (i.e., visual, auditory, even tactile if fingers are placed on a surface) and the respective finger movement coincide in a unique way with the mathematical concept to be learned - the understanding of the quantitative meaning of four.

Moreover, finger use seems to be an intuitive strategy children apply spontaneously at some point during their numeracy development when it comes to composing and decomposing numbers. For instance, reflecting that seven fingers can be composed of the thumb and the index finger on one hand and all five fingers of the other hand, etc., seems a first step to master arithmetic operations^13,14^. Recent research substantiated this claim, by indicating that using fingers to solve initial calculations is beneficial^15,16^, particularly from kindergarten up to mid of grade 2^14^. Furthermore, early numeracy interventions using fingers demonstrated significant beneficial outcomes^12,17–21^. Ollivier et al.^19^, for instance, implemented an intensive 10-week training program for 5- to 6-year-old children with five training sessions per week lasting 45 min each. The intervention combined finger-based numeracy activities with perceptual and motor training using fingers and hands compared to a business-as-usual control group. Despite promising results regarding the development of basic arithmetic skills, the study was limited by its small sample size (*n* = 36). This limitation was overcome by Frey et al.^17^, who also implemented an extensive 18 session intervention (25–30 min each) during regular math lessons with 242 students during first grade (mostly 6- to 7-year-olds) and compared its effects to a control group that followed standard mathematics instruction. The training included aspects of finger gnosis as a foundational skill, helping children develop a better sense of finger identification and differentiation. Nevertheless, children primarily practiced finger number associations as well as finger-based arithmetic. Results indicated that the finger-based intervention significantly improved children’s subtraction and addition skills compared to the control group from the start to the end of first and until the middle of second grade. An adaptation of this finger-based intervention for preschoolers (ages 5–6), however, did not yield similar effects^22^.

These results illustrate that finger-based early numeracy interventions are not per se effective and that the specific contribution of different intervention components such as fine motor skills and finger gnosis to different numeracy skills is still unclear as most interventions included multiple components. Thus, intervention studies that focus on individual components may be more conclusive. For instance, the study by Orrantia et al.^11^, investigated cardinality understanding in 3-year-olds and found that children from a finger-based intervention group outperformed those in the control group who received a similar intervention but without finger number gesturing. Similar beneficial results were observed for finger-based interventions focusing on preschoolers’ arithmetic skills only^20,23^.

In summary, most existing interventions took one of two approaches: they either focused narrowly on specific early numeracy skills^11,20^, or they addressed a broader range of fine-motor and numerical skills^17,18,22^. With the first approach, it is possible to make very specific statements about the effect of finger use on learning a particular numeracy skill. However, what remains unclear is how effective the intervention is for children who, regardless of finger use, do not yet possess the necessary numerical prerequisites to understand that. With the second approach, the actual impact of the promotion of fine motor skills and finger gnosis remains unclear. While positive correlations between fine motor abilities or finger gnosis and numeracy have consistently been found (see Barrocas et al.^24^ for a review), findings by Lewis and Weixler^25^ suggest that dedicating instructional time to fine motor activities does not enhance but may even hinder mathematics achievement in kindergarten, considering the limited amount of instructional time available.

Against this background, the question arises, (1) whether a comprehensive finger-based interventions targeting not only a specific early numeracy skill but rather aiming at systematically and progressively developing early numeracy skills, are more or similarly effective than a more isolated intervention and (2) whether a finger-based numeracy intervention not explicitly promoting domain-general skills such as fine motor skills and finger gnosia is also effective.

To address these questions, the present study aimed to develop and evaluate a finger-based early numeracy intervention. This intervention was specifically designed to (i) systematically follow early numeracy development while using fingers as primary and embodied manipulative to count, depict magnitudes, and perform basic arithmetic (ii) with no intervention activities on more domain-general abilities such as fine motor or finger sensory skills. This approach seeks to provide clarity on the efficacy of a finger-based intervention for early numeracy development. In the following, we will discuss theoretical considerations on how finger use is argued to foster early numeracy development.

## Finger use and early numeracy development

When it comes to using fingers in early numeracy contexts, there are basically two different aspects to be distinguished from a motor perspective (see also Neveu et al.^26^). On the one hand, there is finger counting, which means that single fingers are extended successively one after another while each finger is assigned to exactly one number (word) in stable order (e.g., thumb for one, index finger for two etc. as found in Continental European cultures; see Bender & Beller^27^ for an overview of cultural differences). On the other hand, there is finger number gesturing also known as finger montring^28^, which reflects that fingers are extended simultaneously to represent a specific quantity (e.g., thumb and index finger to represent the quantity of two). 3- to 5-year-olds were shown to be more proficient in finger counting compared to finger number gesturing suggesting a developmental trajectory from finger counting to gesturing^29^. When it comes to basic arithmetic, children typically use both finger counting and finger number gestures, depending on the strategy they choose. For instance, when solving an addition problem like 4 + 2, they might first display each number using finger gestures on separate hands and then count the total number of extended fingers (COUNT-ALL strategy^30^. Thus, it is no surprise that both aspects play an important role in the development of basic arithmetic skills^29,31^. Taking into account all different aspects of finger use, Roesch and Moeller^32^ summarized theoretical arguments on why and how fingers might be useful to support early numeracy at different stages, following the developmental model proposed by Krajewski and Schneider^8^. The latter assume early numeracy to develop on three consecutive levels:

At level I, children learn to recite the exact sequence of number words and develop counting skills. From a theoretical perspective, there are multiple aspects of how finger use may support this process. When counting with fingers, each finger is assigned to one specific number (e.g., thumb for one, index finger for two), making the counting principle of one-to-one-correspondence easily understandable^33^. But also the acquisition of number words themselves may be supported through finger counting, as the association between fingers and numbers can help perceive number words as distinct phonological units^34^ and may serve as a kind of marker that facilitates their memorization^33,35^. Furthermore, the counting principle of stable order as well as the ordinal concept of numbers are conveyed by finger counting habits as fingers are always counted in the same order establishing an ordered sequence of fingers^33,35^. This should, for instance, support the understanding of predecessors and successors (e.g., “ten” — linked to the final finger counted — comes after “nine,” linked to the preceding one). However, conceptual knowledge of counting principles does not necessarily develop naturally through procedural finger counting when solving addition problems. For example, Krenger and Thevenot^36^ observed that finger users generally showed better understanding of counting principles than non-finger users. However, some children who used their fingers did not understand these principles, which was associated with lower addition performance. Importantly, these results do not imply that finger counting lacks the potential to support acquiring the correct sequence of number words and learning about conceptual counting principles. Instead, they suggest that explicit instruction focusing on counting itself, rather than on solving additions through counting, is needed to fully grasp counting principles.

On level II of the developmental model by Krajewski and Schneider^8^, children become aware of the cardinal meaning conveyed by each number word. During finger counting, fingers are stretched out one after another while extended ones are not pulled in again. This procedure has also been termed “cardinalized counting”^33^, as it allows for linking each number word to the corresponding quantity. However, fingers offer an additional advantage: Since they are always extended in the same sequence when counting, each number is not only associated with a specific finger but also with a predefined combination of extended fingers - the respective finger number gesture. Similar to dot patterns on dice, these finger patterns develop iconic character, making them easy to recognize^37–39^ and recreate by quickly extending the corresponding number of fingers simultaneously. When these fingers are placed on a surface and the action is accompanied by verbalization, sensory (i.e., tactile, visual, auditory) and motor processes are uniquely aligned, which might facilitate the understanding of the cardinal meaning of numbers (see Jordan & Baker^40^ for the impact of multisensory information on numerical learning). Thus, it seems reasonable that especially finger number gesturing promotes cardinal number understanding, a claim that has already been successfully examined empirically in the meantime^11^.

At level III of their developmental model, Krajewski and Schneider^41^ suggest that children begin to understand relations between numbers. This understanding allows them to compose (e.g., 5 and 2 is 7) and decompose (e.g., 7 comprises 5 and 2) numbers and to quantify the exact difference between two numbers (e.g., 7 is 2 more than 5). The arrangement of the fingers on two hands provides a natural structure illustrating number relations, in particular for numbers 5 and 10. As each finger number gesture consists of a specific number of extended and folded fingers on each hand (e.g., 3 is represented by three extended and two unfolded fingers on one hand as well as another five unfolded fingers on the other hand), there is an inherent structure in every finger number gesture, visualizing part-whole relations for 5 and 10 (e.g., 3–2–5 and 3–7–10).Relations of other quantities can also be visualized using fingers when the focus is on the extended fingers only. For example, seven extended fingers can easily be broken down by grouping them into five and two or four and three (e.g., by placing a pencil as a separator between the subsets). Finally, recognizing and utilizing these structures is assumed to allow children to combine quantities and solve basic arithmetic problems efficiently using their fingers. When children are familiar with finger number gestures, they can solve addition problems like 4 + 2 by first forming the finger number gesture of four, then adding two fingers following the finger counting sequence, resulting in the sum – the finger number gesture of six, which they may even recognize without counting. A procedure like that can therefore be described as structure-based finger arithmetic.

In principle, there may be different approaches through which children can gain insights into mathematical concepts such as cardinality, number (de-)composition etc. through using their fingers, and we presented only some examples of what might be possible. Moreover, it should be noted that we do not intend to give the impression that mastery of numeracy concepts develops automatically just by involving embodied procedures. Rather, we assume that these concepts build on each other, as described in different developmental models, and that finger use can serve as a meaningful and multisensory tool facilitating procedural processes and offering conceptual insights. Most finger-based early numeracy interventions either focused narrowly on specific numeracy skills^11,20^, or they addressed a broader range of skills including domain-general aspects such as fine motor skills or finger gnosia^17,18,22^. Therefore, it remains unclear whether a systematic and focused numeracy intervention using fingers is effective at the kindergarten age.

### The present study

To address this research gap, we developed and evaluated a finger-based numeracy intervention specifically designed to systematically follow early numeracy development. The program consisted of 12 sessions (30 min each) targeted at enhancing children’s counting skills (level I), cardinality understanding and ordering (level II), and basic arithmetic skills (level III) through finger counting, finger number gesturing, and structure-based finger arithmetic, while no content was included that exclusively targeted more domain-general skills such as fine motor and finger sensory skills. Subsequently, we evaluated the program in an intervention study using a pre-posttest design comparing the intervention group to a business-as-usual control group.

Reflecting the theoretical discussion above, we hypothesized the intervention group to outperform the control group at posttest with respect to their overall numeracy skills as well as separately on all three developmental levels suggested by Krajewski and Schneider^8^. To ensure that potential intervention effects are not driven by pre-test differences between groups on numeracy but also domain-general skills such as fluid reasoning and working memory we considered these as covariates. Moreover, we hypothesized the intervention effects to be specific to the content of the intervention (i.e., numeracy skills) and expected no significant performance differences at posttest with respect to fluid reasoning and spatial working memory. We will therefore include domain-general skills such as fluid reasoning and working memory not only as covariates, but also as predicted outcome variables in our analyses. Finally, we were interested in the development and characteristics of finger use as a consequence of the intervention as well as in general. In particular we expected the finger-based intervention to lead to a higher number of finger users in arithmetic tasks at posttest compared to the control group and that finger users (irrespective of group assignment) should outperform non-finger users with respect to early numeracy at both measurement time points^15^.

## Method

### Participants

In total, *n* = 89 children from eight childcare facilities located in two different towns in southern Germany participated in this study. In the German education system, children usually spend three years in childcare (from around the age of three to six years) before entering elementary school. For this study, only children in their last year, about 5 months before school entry, were asked to participate. All children had no or limited prior exposure to formal mathematics instruction as there is no mandatory mathematics curriculum for early years in Germany.

Data of *n* = 2 children were excluded from analyses, as no posttest results were available. Moreover, another *n* = 17 children were excluded from analyses, as they scored at ceiling already at pretest (i.e., accuracy ≥ 85%, for details see results section). Thus, the final sample consisted of *n* = 70 5- to 6-year-olds, of which *n* = 33 belonged to the intervention group (20 girls, 13 boys; *M*_*age*_=6.16, *SD* = 0.28) and *n* = 37 to the control group (20 girls, 17 boys; *M*_*age*_=6.14, *SD* = 0.38). The majority of children spoke German as their first language (*n* = 43; *n* = 8 other first language; *n* = 19 missing information), which corresponds to 52% of the intervention group and 70% of the control group. As highest school-leaving qualification, most mothers (*n* = 35, *n* = 19 missing) as well as fathers (*n* = 33, *n* = 21 missing) indicated at least a university entrance qualification, which corresponds to 16 mothers and 14 fathers of the intervention group (control group: 19 mothers and 19 fathers).

### Procedure

After childcare facilities agreed to participate and the consent of the respective provider was granted, they were assigned to either the experimental or the control group. Group assignment was made in a way to ensure that kindergartens from both locations (i.e., the two towns in southern Germany) were represented in each group, and that the number of children per group was approximately equivalent. Subsequently, the pretest was conducted by trained student assistants in the respective facilities (one individual testing session per child of about 30 min). This procedure did not allow for exactly equal group sizes in the intervention and control group (i.e., *n* = 33 vs. *n* = 37). Next, the children of the intervention group received 12 sessions of a finger-based numeracy intervention (i.e., two 30-minute sessions per week; holidays excluded) conducted in small groups of up to six children by the first author. The children of the control group did not receive any additional numeracy training during this period. Afterwards, the posttest was done following an identical procedure as the pretest. To minimize time effects, both assessment periods were kept as short as possible (pretest: 24 days, posttest: 25 days). All interviewers were blinded to the conditions in both pre- and posttest and the researcher conducting the intervention was not involved at post-testing. After the intervention, a workshop on early numeracy development and finger use was offered to all participating childcare facilities and all intervention materials were provided free of charge. Children participated only after written informed consent was obtained from legal guardians. Additionally, oral assents were obtained from children prior to testing. The study was approved by the local research ethics committee of the University of Tuebingen, Faculty of Economics and Social Sciences. All research was performed in accordance with the Declaration of Helsinki.

### Tasks

In pre- and posttest, children’s early numeracy as well as domain-general skills such as spatial working memory and fluid reasoning were assessed.

#### Early numeracy

We used the MBK-0^42^ to assess early numeracy on the three developmental levels proposed by Krajewski and Schneider^8^. This involved all subtests of the short version plus one of the long version of this test. In addition, we also administered two subtests of the TEDI-MATH^43,44^ assessing symbolic addition and subtraction, as this task type is typically used for measuring finger use^15,20^. To evaluate interventions effects, we calculated an overall early numeracy score over all tasks described below as well as one score each for level I, II, and III. Reliability of the overall early numeracy score based on Cronbach’s alpha was high with 0.93 both at pre- and at posttest.

### Level I: Numbers and counting

To assess children’s level I numeracy skills, the MBK-0^42^ provides two subscales. In the subscale *Number Sequence*, children have to count as far as possible with the maximum score of four points awarded for counting correctly up to at least 31 (3 points for counting up to a number between 21 and 30, 2 points for counting up to a number between 11 and 20, 1 point for counting up to a number between 1 and10). Furthermore, children have to name successor (3 items/points) and predecessor numbers (3 items/points) within the number range up to 20, and to count backwards starting from number five (2 points for correct backward counting, 1 point if at least two numbers are counted correctly), resulting in a maximum score of 12 points for the subscale. In the subscale *Digit Knowledge*, numbers 1 to 20 are shown to children on paper cards in mixed order (i.e., one number per card) and children are asked to name each number correctly (max. 20 points). The sum score of both tasks (max. 32 points) was considered in the analyses. Reliability based on Cronbach’s alpha was 0.93 at pretest and 0.91 at posttest.

### Level II: Cardinality understanding and ordering

Level II numeracy skills were assessed using three subscales of the MBK-0^42^. In the task *Number Concept*, children have to match a given digit from 1 to 10 to a card showing the corresponding quantity out of a selection of distractor cards (3 items/points). The task then is reversed, and children are asked to match a given quantity to the correct digit (2 items/points). A total of up to five points can be achieved on this subscale. In the subscale *Number Seriation*, children are presented with a series of dotted ladybugs (black drawings on a yellow background) arranged by the number of dots on their back (one to eight in ascending left-to-right-order), with one ladybug missing in each row. Children have to identify the missing ladybug from a selection (3 items/points). Additionally, they are asked to identify the ladybug with the number of dots the same as their age and state which ladybugs are younger and older than this one (2 points each for correctly naming all ladybugs and 1 point for naming only some correctly). A total of up to seven points can be achieved on this subscale. In *Number Comparison*, children are orally presented with pairs of numbers within the range up to 10 and subsequently up to 20 and have to indicate the larger or smaller number. A total of up to eight points can be achieved on this subscale. A sum score across all three subscales was used in the analyses (max. 20 points). Cronbach’s Alpha was 0.70 at pretest and 0.74 at posttest.

### Level III: Basic arithmetic

Level III numeracy skills were assessed using three subscales. In the subscale *Word Problems* (MBK-0^42^), children have to solve six orally presented arithmetic word problems (i.e., combine, change, compare, equalize problems) illustrated by a corresponding number of glass stones (e.g. “You have 4 glass stones - interviewer puts down four green glass stones in front of the child in random order. I have 5 glass stones - interviewer puts down five blue glass stones in front of himself/herself. How many do we have together?”). A total of up to six points can be achieved on this subscale. In the subscale *Symbolic addition* of the TEDI-MATH^43^, children are shown and read aloud symbolic addition problems presented on paper cards (one item per card). We only used the first 10 items from the original subscale including single-digit problems with solutions within the number range up to 15. A total of up to 10 points can be achieved on this subscale. In the subscale *Symbolic subtraction* of the TEDI-MATH^43^, symbolic subtraction problems are presented and read aloud the same way as in the addition task. We only considered the first six items from the original subscale, including problems within the number range up to 10. Thus, a maximum of up to six points can be awarded on this subscale. Both the *symbolic addition* and *subtraction* task were discontinued as soon as three consecutive items were answered incorrectly or left unanswered. A sum score was calculated (max. 22 points) and used in subsequent analyses (Cronbach’s alpha 0.83 at pretest and 0.87 at posttest). Across all three subscales, interviewers noted whether children used their fingers to solve an item.

### Fluid reasoning

Children’s fluid reasoning ability was assessed using subset B (12 items) of the Colored Progressive Matrices (CPM^45^. In this matrices test, colored patterns with a missing patch are shown to the children, who have to identify the missing patch out of six probe patterns. The number of correctly solved items was considered in the analyses (Cronbach’s alpha 0.49 at pretest and 0.59 at posttest).

### Spatial working memory

Children’s spatial working memory was assessed using an adapted version of the Corsi Block-Tapping Task^46^. The interviewer tapped a number of wooden blocks (i.e., from two to seven blocks) in a specific order on a board with a total of nine blocks. Children then had to tap the blocks in the same order in the first half of the subtest and in reverse order in the second half of the subtest. Both parts consisted of up to 12 items, with two sequences of the same length each. Both the forward and backward subscale were discontinued as soon as the children repeated both sequences of the same length incorrectly. The number of correctly reproduced sequences was considered in the analyses (max. 24 points; Cronbach’s Alpha 0.63 at pretest and 0.67 at posttest).

### Description of the finger-based early numeracy intervention

**Fig. 1:**
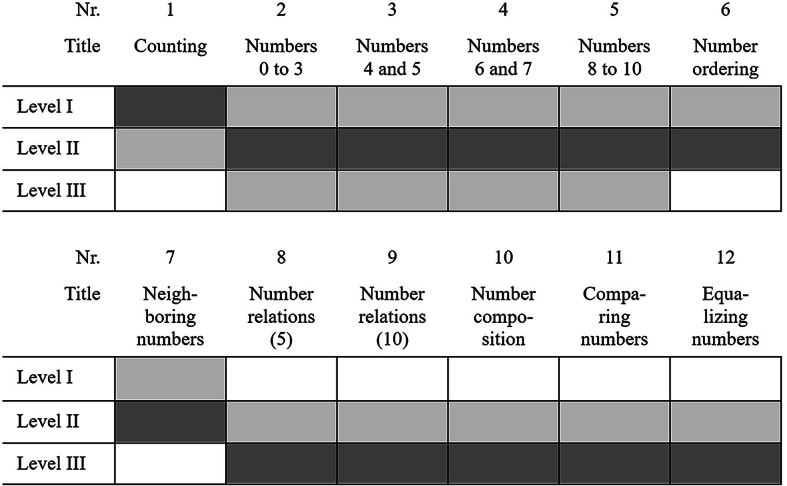
Overview of the sequence of sessions and respective levels addressed in each session. The level primarily focused on in a session is reflected in dark grey, while levels also addressed – even though not the primary focus of the session – are shown in light grey.

The finger-based numeracy intervention was developed to promote early numeracy skills on all three levels of the model proposed by Krajewski and Schneider^8^ through systematic development of finger use as outlined in the introduction (see Fig. 1 for an overview of the sequence of sessions and connections to the respective levels).

Accordingly, finger counting built the basis and, was successively (i.e., starting from sessions 4) developed into a simultaneous extension of fingers to represent cardinality (finger number gesturing), and subsequent structure-based finger arithmetic (i.e., starting from sessions 8). The latter was to illustrate part-whole relations, number differences, and simple addition problems. To differentiate between counting and cardinality, all finger activities for counting were done palm up (i.e., the natural finger counting posture). In contrast, hands were turned palm down and placed on a surface (i.e., the table children set around) when the final number was reached to emphasize the cardinality aspect (e.g., thumb “one”, index finger “two”, middle finger “three”; turning the hands palm down on the tabletop “It’s three”).

As soon as children were familiar with the finger number gestures, they were encouraged to skip the counting process, extend the respective number of fingers simultaneously, and place them palm down on the tabletop. Moreover, to visualize the whole structure of a finger number gesture, it was always both hands that were used for counting/cardinality representation, irrespective of whether the number was smaller or larger than five. Throughout the 12 sessions, the fingers were used as a central, though not exclusive, manipulative, meaning that they were involved in every session, but were not necessarily used in every single activity within a unit. Numbers worked on ranged between 0 and 10. All activities were conducted in small groups of up to six children and led by the first author, who provided feedback and support to children. Finger-based strategies to solve arithmetic problems were explicitly introduced to children. Subsequently, the procedure was practiced together within the respective activities several times. As practice continued, children were still encouraged to use their fingers when needed to help model a task. However, children who were able to solve a task mentally, or who were generally already capable of mental arithmetic, were not required to use their fingers.

The intervention was accompanied by two puppets called “Ed and Ted” (i.e., gloves embroidered with faces) which were worn by the instructor at the beginning and end of each session. At the beginning, Ed and Ted greeted the children and sang an introductory song together with them. They typically introduced the topic of the session, for example, by describing an observation or an argument they had. Following this, one or two playful exercises were carried out. During these activities, Ed and Ted stepped back as “silent observers” before reappearing at the end of the session – either for a concluding game or to say goodbye. To provide an impression of how the individual sessions were conducted, the supplementary materials include, as an example, the detailed instructions for two of the 12 sessions (i.e., sessions 4 and 9). Overall, the following activities were conducted at least once over the course of the 12 sessions:

- *Counting principles* (session 1): Count to 10 with fingers and count glass stones; understand the principles of one-to-one-correspondence and order invariance (level I).
- *How many?* (session 1): Determine the number of claps, hops, etc. by finger counting (levels I and II).
- *Numbers and finger number gestures* (sessions 2 to 5): Count to a number between 0 and 10 (levels I and II) and describe the structure of the resulting finger number gesture (i.e., folded and extended fingers on both hands, level III); match the finger number gesture with given finger pictures and digit cards and sort in ascending order (level II). Session 2 focused on numbers 0–3, session 3 on numbers 4 and 5, session 4 on numbers 6 and 7, and session 5 on numbers 8–10. Smaller numbers that were introduced previously were consistently included when ordering finger pictures and digit cards.
- *Finger-number booklet* (sessions 2 to 5): Recognize and describe the visual characteristics of the digits by tracing each digit in the booklet with writing the direction indicated by a road and a car; create own finger pictures (i.e., painted finger number gestures) by drawing along the own finger number gesture placed at a booklet page (level II).
- *Blindfold game with numbers* (sessions 2 to 5): Identify with closed eyes which already introduced digit is traced with the own finger led by either Ed and Ted or another child. In sessions 4 and 5 children play the game in pairs of two, and the child, whose finger is guided with eyes closed, must display the guessed number using a finger number gesture (level II).
- *Eagle-eye game* (session 4 and 5): Rapid identification and mapping of already introduced finger picture cards to digit cards (level II).
- *Number houses* (session 6 and 7): Work with houses for numbers 0 to 10 (i.e., contours of houses, arranged in ascending left-to-right-order, and stuck to the ground with tape); place the correct number of glass stones, blueberry picture cards, finger picture cards, and digit cards into each house by estimating and counting the quantities (level I); identify more/less and describe how the numbers change in ascending order (level II); figure out – based on quantities – to which number an emptied house in the row belongs (level II).
- *Number path* (session 7): Walk the number path (i.e., a line taped to the ground in front of the number houses) forward/backward and count while passing the houses; position oneself in front of a house and identify neighboring houses based on the quantities (levels I and II).
- *Neighboring numbers* (session 8): Roll the dice (with digits 0 to 9), do the correct finger gesture, and find the two neighboring numbers using fingers (level II).
- *The small and the large number caterpillar* (sessions 8 to 10): As described in Björklund et al.^12^, children work with the small and large number caterpillar (i.e., a string of 5 green pearls for the small caterpillar and 10 green/blue pearls for the large caterpillar that are partly hidden under a fabric leaf; see also Kullberg et al.^23^); built the finger number gesture corresponding to the visible pearls (level II) and identify the number of hidden pearls corresponding to the number of unfolded fingers (level III).
- *Number domino* (session 9): Play number domino while only the number that adds up to 10 is allowed to be placed next to the number already shown; apply the same finger strategy as in the caterpillar game to identify which number adds up to 10 (levels II and III).
- *Ed and Ted’s treasure chest* (session 9): All children play together against Ed and Ted by rolling a dice (with digits 0 to 9) to win the respective number of glass stones from a treasure chest with 10 glass stones inside. The number rolled on the dice indicates how many glass stones the children receive in a round. They calculate the number of glass stones that remain within Ed and Ted’s treasure chest using their fingers, compare who won more (i.e., Ed & Ted or them) and award one point to the winner (levels II and III). The first to reach 6 points wins.
- *Ed and Ted’s treasure chest advanced* (sessions 10 and 11): Learn how to add numbers using structure-based finger arithmetic (see description above); all children play together against Ed and Ted. Now, they roll two dices (with digits 0 to 5) and add the numbers applying structure-based finger arithmetic to identify how many glass stones they receive in a round. They calculate the number of glass stones that remain within Ed and Ted’s treasure chest using their fingers, compare who won more (i.e., Ed & Ted or them) and award one point to the winner (levels II and III). The first to reach 6 points wins.
- *Word problems* (sessions 8 and 12): Ed and Ted each have a bowl with different numbers of snacks; children determine how many more snacks Ed needs to match Ted’s number of snacks by using fingers (levels II and III).
- *Team Ed vs. Team Ted – Who has more?* (sessions 11 and 12): Children play in two teams. Each team has a set of number cards (from 0 to 10) laid out openly at the table. The first team selects one card from each set, determines the larger number (level II), and calculates the difference between both numbers by using their fingers (level III). The difference is paid out in glass stones, while in the end the team with the most glass stones wins.

### Data analysis

First, we examined pretest performance, excluding children scoring at ceiling (i.e., overall accuracy for early numeracy ≥ 85%) as there were no measurable intervention effects to be expected for these. Subsequently, we checked all outcome and control variables for baseline differences between intervention and control group using *t*-tests for independent groups. According to WWC standards^47^, it is recommended to statistically adjust for minor pretest differences ranging between *d*=0.05 and 0.25 to assure baseline equivalence, irrespective of whether or not differences turn significant. Accordingly, all variables that met this criterion were considered as control variables in the following analyses. To evaluate treatment effects, analyses of covariance were conducted with posttest performance in the respective domain as the outcome variable, group as fixed factor (intervention vs. control group) and the respective pretest performance as well as control variables as covariates.

To evaluate the development and characteristics of finger use, we replicated the procedure reported by Krenger and Thevenot^15^. Based on observations during arithmetic tasks at pre- and posttest, we created two groups: finger-users (i.e., children who used finger-based strategies at least once to solve a task) and non-finger users (i.e., children who never used finger-based strategies). Differences between these groups with respect to early numeracy were then evaluated using independent sample *t*-tests. Data was analyzed using SPSS Version 28.0.1.0.

## Results

In the first part of this section, we will report on the results of the efficacy and specificity of the finger-based early numeracy intervention . Subsequently, we will provide detailed analyses of potential intervention effects on the different levels of early numeracy development (i.e., level I to III according to the developmental model by Krajewski & Schneider^8^), followed by an analysis regarding the development and characteristics of finger users in arithmetic tasks as a consequence of the training and in the whole sample.

### Efficacy of the intervention to improve early numeracy

In a first step, we looked at pretest performance on the individual as well as group level (i.e., intervention vs. control group). Inspection of individual pretest performance revealed that *n* = 17 children (intervention group: *n* = 12; control group: *n* = 5) scored at ceiling already at pretest with accuracy scores on early numeracy of ≥ 85% (*M* = 91.10%, *SD* = 4.41). As no further (at least measurable) improvement with respect to early numeracy was to be expected for these children, the data of these children were excluded from the following analyses.

Second, we evaluated potential pretest differences between intervention and control group. As can be seen from the descriptives depicted in Table 1, the intervention group outperformed the control group at pretest both with respect to early numeracy and the control variables (i.e., spatial working memory and fluid reasoning).

**Table 1:** Descriptives of the primary outcome and the control variables at pre- and posttest.

		Control group (*N* = 37)		Intervention group (*N* = 33)			
	Max	M	SD	M	SD	Cohen‘s d	*p*
Early numeracy skills (t1)	74	42.87	12.57	45.72	11.17	0.24	0.320
Spatial working memory (t1)	24	7.41	2.10	8.70	2.17	0.61	0.014
Fluid reasoning (t1)	12	5.05	1.83	5.55	1.66	0.28	0.245
Early numeracy skills (t2)	74	45.62	12.24	50.97	11.38	0.45	---
Spatial working memory (t2)	24	8.86	2.31	9.64	2.43	0.33	---
Fluid reasoning (t2)	12	5.76	1.92	5.97	1.86	0.11	---

Note. Max=Maximum score points to be achieved on the respective scale. Cohen’s *d* and *p*-values refer to between-group differences.

For the primary outcome early numeracy (*d*=0.24) as well as for fluid reasoning (*d*=0.28), pretest differences turned out to be non-significant, *t*(68) = 1.00, *p*=.320 and *t*(68) = 1.17, *p*=.245, of small size according to Cohen^48^, and acceptable after adjustment by inclusion as covariates in the following analyses. However, significant medium sized pretest differences occurred for spatial working memory, *t*(68) = 2.53, *p*=.014, *d*=0.61, while regression slopes were comparable between groups as indicated by non-significant interactions between group (intervention group vs. control group) and control variables (early numeracy skills, fluid reasoning, and spatial working memory). Nonetheless, this means that both groups might not be fully comparable, at least with respect to spatial working memory. Thus, we first ran an ANCOVA with early numeracy skills (t2) as outcome including only early numeracy skills (t1) and fluid reasoning (t1) as covariates, adding spatial working memory (t1) afterwards as additional covariate in a second model to estimate treatment effects reliably. Results revealed that only early numeracy (t1) and group, but not fluid reasoning (t1) significantly contributed to explaining posttest performance in early numeracy (see Table 2).

**Table 2:** Results of ANCOVA for posttest outcomes early numeracy (t2), fluid reasoning (t2), and spatial working memory (t2).

	Model I			Model II		
	F(1,66)	*p*	η_*p*_^2^	F(1,65)	*p*	η_*p*_^2^
**Early numeracy (t2)**						
Group	4.68	0.034	0.07*	3.84	0.054	0.06^+^
Early numeracy (t1)	256.32	< 0.001	0.80**	236.10	< 0.001	0.78**
Fluid reasoning (t1)	0.52	0.473	0.01	0.25	0.620	0.00
Spatial working memory (t1)	---	---	---	0.24	0.624	0.00
**Fluid reasoning (t2)**						
Group	0.02	0.884	0.00	0.27	0.603	0.00
Early numeracy (t1)	1.55	0.218	0.02	0.75	0.389	0.01
Fluid reasoning (t1)	11.34	0.001	0.15**	7.00	0.010	0.10*
Spatial working memory (t1)	---	---	---	2.32	0.133	0.03
**Spatial working memory (t2)**						
Group	---	---	---	0.00	0.949	0.00
Early numeracy (t1)	---	---	---	1.40	0.241	0.02
Fluid reasoning (t1)	---	---	---	0.00	0.969	0.00
Spatial working memory (t1)	---	---	---	19.26	< 0.001	0.23**

Note. Outcome variables in bold. ^**^p<.01, ^*^p<.05, ^+^*p*<.10.

Effect sizes were large for early numeracy skills (t1) and medium for the effect of group referring to Cohen48, with the intervention group significantly outperforming the control group in early numeracy (t2) adjusted for pretest performance and fluid reasoning (intervention group: *M*_*adj.*_=49.56, *SE*=0.90; control group: *M*_*adj.*_=46.88, *SE*=0.85).

When adding spatial working memory as a third covariate to the model, the pattern of results did not change, with only early numeracy (t1) and group (marginally significant *p*=.054), but neither fluid reasoning (t1) nor spatial working memory significantly contributing to explaining posttest performance in early numeracy.

To evaluate whether the intervention improved children’s early numeracy specifically, we ran another ANCOVA with fluid reasoning (t2) as the outcome and fluid reasoning (t1), spatial working memory (t1), and early numeracy (t1) as covariates. Results indicated that only fluid reasoning (t1), but neither spatial working memory (t1), nor early numeracy (t1) or group significantly contributed to explaining posttest performance in fluid reasoning (t2) (see Table 2; intervention group: *M*_*adj.*_=5.83, *SE*=0.30; control group: *M*_*adj.*_=5.89, *SE*=0.28).

A similar pattern occurred for posttest performance in spatial working memory (t2). Only spatial working memory (t1), but neither fluid reasoning (t1) nor early numeracy (t1) nor group significantly explained posttest performance in spatial working memory (t2) (intervention group: *M*_*adj.*_=9.21, *SE*=0.34; control group: *M*_*adj.*_=9.24, *SE*=0.34).

### Intervention effects on specific levels of early numeracy development

To evaluate intervention effects on specific levels of early numeracy skills, we first examined potential pretest differences between intervention and control group on the respective levels.

As can be seen from Table 3, the intervention group outperformed the control group at pretest on all levels of early numeracy (for descriptives of all subscales included in the sum scores for level I, II, and II see supplementary online material). Pretest differences turned out to be non-significant, of small size according to Cohen^48^ ranging between *d*=0.11 and *d*=0.29, and acceptable after adjustment (i.e., inclusion of the respective pretest score as covariate in addition to fluid reasoning and spatial working memory).

**Table 3:** Descriptives of early numeracy level I, II, and III.

	Control group (*N* = 37)			Intervention group (*N* = 33)			
	Max	M	SD	M	SD	Cohen‘s d	*p*
Level I: Counting (t1)	32	21.38	8.24	22.79	6.69	0.19	0.438
Level II: Cardinality (t1)	20	16.11	2.69	16.42	3.22	0.11	0.656
Level III: Basic arithmetic (t1)	22	5.38	4.03	6.52	3.90	0.29	0.236
Level I: Counting (t2)	32	22.32	7.53	25.18	6.13	0.41	---
Level II: Cardinality (t2)	20	16.78	2.81	17.27	3.07	0.17	---
Level III: Basic arithmetic (t2)	22	6.51	4.48	8.52	4.75	0.43	---

Note. Max=Maximum score points to be achieved on the respective scale. Cohen’s *d* and *p*-values refer to between-group differences.

**Table 4:** Results of ANCOVA for the posttest outcomes level I: Counting, level II: Cardinality, level III: Basic arithmetic with pretest performance, fluid reasoning, and spatial working memory as covariates.

	Model I			Model II		
	F(1,66)	*p*	η_*p*_^2^	F(1,65)	*p*	η_*p*_^2^
**Level I: Counting (t2)**						
Group	5.11	0.03	0.07*	4.64	0.04	0.07*
Level I: Counting (t1)	245.37	< 0.001	0.79**	237.31	< 0.001	0.79**
Fluid reasoning (t1)	0.00	0.98	0.00	0.00	0.998	0.00
Spatial working memory (t1)	---	---	---	0.00	0.95	0.00
**Level II: Cardinality (t2)**						
Group	0.13	0.72	0.00	0.03	0.87	0.00
Level II: Cardinality (t1)	24.17	< 0.001	0.27**	20.99	< 0.001	0.24**
Fluid reasoning (t1)	1.71	0.20	0.03	0.88	0.35	0.01
Spatial working memory (t1)	---	---	---	0.48	0.49	0.01
**Level III: Basic arithmetic (t2)**						
Group	3.62	0.06	0.05^+^	2.53	0.12	0.04
Level III: Basic arithmetic (t1)	103.20	< 0.001	0.61**	91.31	< 0.001	0.58**
Fluid reasoning (t1)	2.17	0.15	0.03	0.93	0.34	0.01
Spatial working memory (t1)	---	---	---	1.17	0.28	0.02

Note. Outcome variables in bold. ^**^p<.01, ^*^p<.05, ^+^*p*<.10.

As regards effects on posttest number and counting skills (level I), ANCOVA results indicated that only number and counting skills (t1) and group, but not fluid reasoning (t1) contributed significantly (see Table 4). The effect size for group was medium according to Cohen^48^, with the intervention group significantly outperforming the control group in posttest number and counting skills adjusted for pretest performance and fluid reasoning (intervention group: *M*_*adj.*_=24.57, *SE*=0.54; control group: *M*_*adj.*_=22.87, *SE*=0.51). Adding spatial working memory (t1) as further covariate to the model did not change the pattern of results with only number and counting skills (t1) and group significantly explaining variance in posttest counting performance.

For performance on cardinality understanding and ordering (level II), there were no significant group differences at posttest between intervention and control group with cardinality understanding and ordering (t1) being the only significant covariate. The intervention group did not significantly outperform the control group in posttest cardinality understanding and ordering adjusted for pretest performance and fluid reasoning (intervention group: *M*_*adj.*_=17.13, *SE*=0.43; control group: *M*_*adj.*_=16.91, *SE*=0.41). When spatial working memory (t1) was added as covariate, results did not change with still only cardinality understanding and ordering (t1) contributing significantly to cardinality understanding and ordering (t2). As depicted in Table 3, children already scored very high for cardinality understanding and ordering at pretest (*M* = 81.29% accuracy, *SD* = 14.66) and even more so at posttest (*M* = 85.07% accuracy, *SD* = 14.63). As suggested by an anonymous reviewer, we ran an additional analysis excluding all children scoring both ≥ 85% on overall early numeracy and ≥ 85% on cardinality understanding and ordering (level II) to rule out that ceiling effects may have influenced these results. This resulted in a subsample of *n* = 33 children (*n* = 15 intervention group, *n* = 18 control group). However, there was still no intervention effect for cardinality understanding and ordering (t2) as the control group (*M* = 16.22, *SD* = 2.97) even scored a bit better than the intervention group (*M* = 15.40, *SD* = 3.31) in posttest level II skills, besides only marginal pretest differences in cardinality understanding and ordering (*d*=-0.05 in favor of the control group). Thus, ceiling effects alone cannot explain the results.

**Fig. 2:**
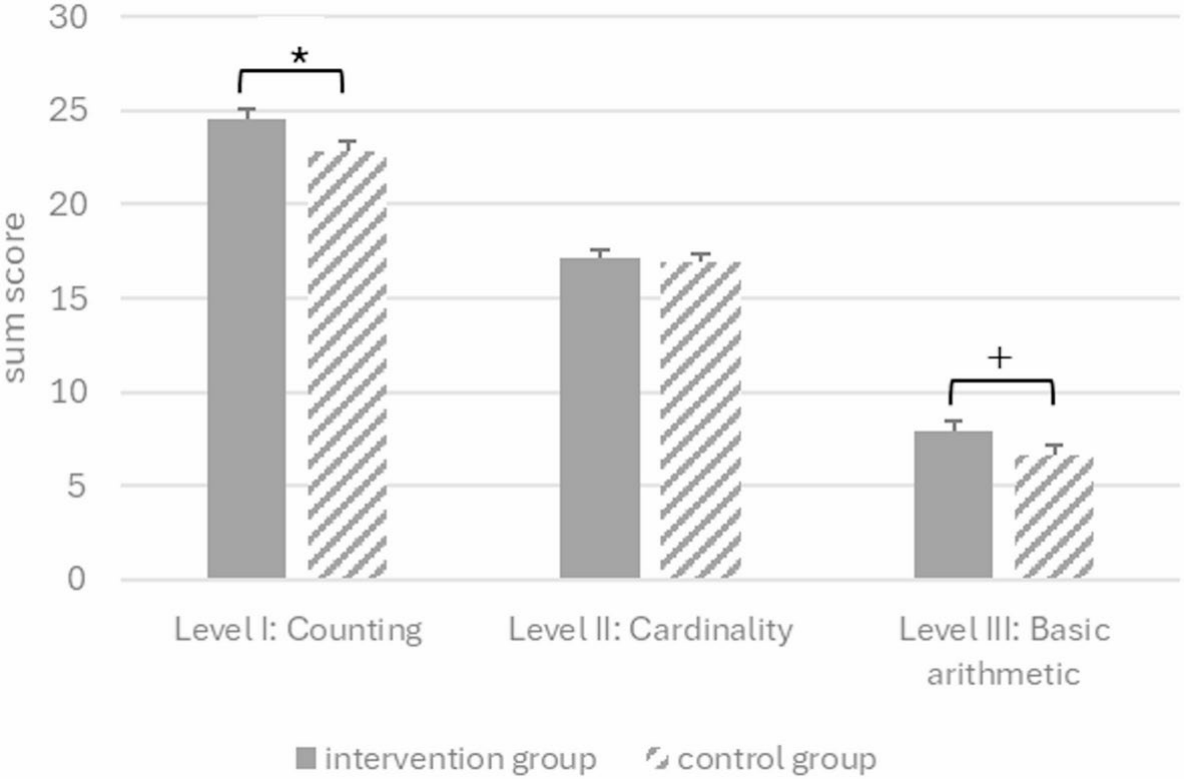
Overview of intervention effects from model I (i.e., mean posttest scores adjusted for pretest performance and fluid reasoning); *p<.05; +*p*<.10; maximum sum score to be achieved on Level I: Counting is 32, Level II: Cardinality is 20, Level III: Basic arithmetic is 22; error bars represent standard errors.

Regarding posttest performance on basic arithmetic (level III), there were marginally significant group differences between intervention and control group. However, basic arithmetic (t1) was the only significant covariate. The effect size for group differences was small according to Cohen^48^, with a tendency for the intervention group outperforming the control group in posttest basic arithmetic skills adjusted for pretest performance and fluid reasoning (intervention group: *M*_*adj.*_=7.92, *SE*=0.48; control group: *M*_*adj.*_=6.67, *SE*=0.45). Considering spatial working memory (t1) in the model resulted in the group difference no longer being marginally significant. In this case, only pretest performance in basic arithmetic contributed significantly. For an overview of the results on the levels I to III see Fig. 2.

### Development of finger use

**Fig. 3:**
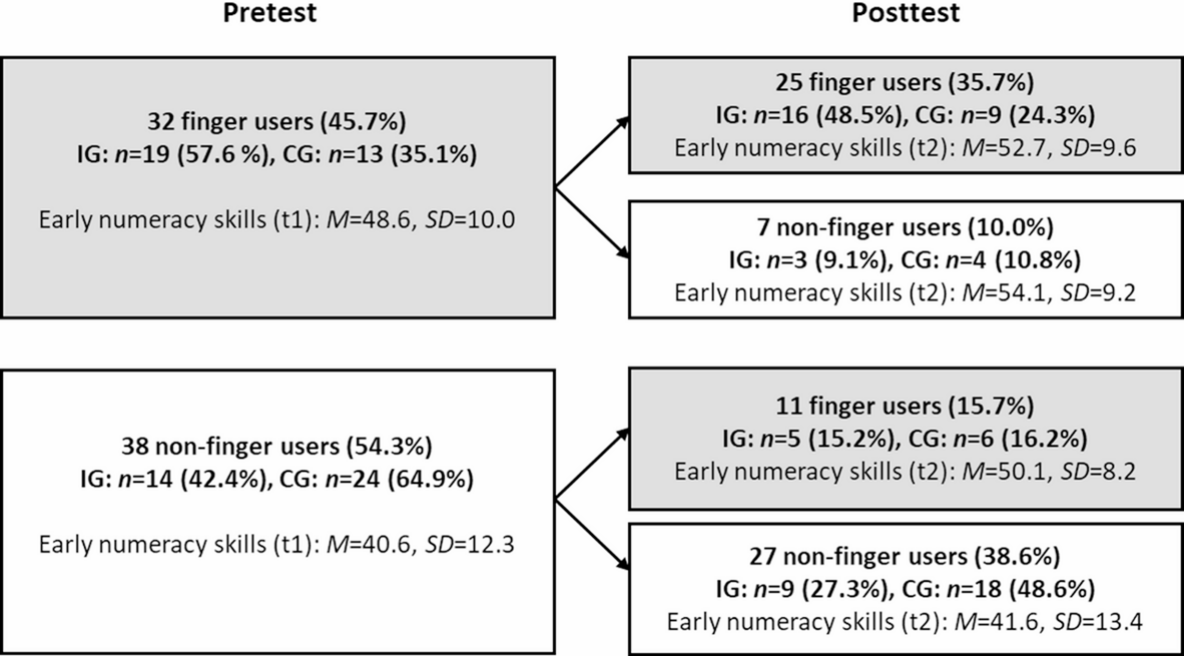
Number of finger users and non-finger users at pre- and posttest and their mean performance in early numeracy. In parentheses the proportion of finger users and non-finger-users in the whole sample, as well as within the intervention group (IG) and the control group (CG).

To evaluate the development of finger use in the control and intervention group, we replicated the procedure reported by Krenger and Thevenot^15^. Based on observations during the three basic arithmetic subscales (i.e., word problems, symbolic addition, symbolic subtraction), we created groups of finger users and non-finger users both at pretest as well as at posttest. At pretest, 32 children (45.7%) were classified as finger users and 38 children (54.3%) as non-finger users (see Fig. 3). The highest proportion of finger users was observed in the symbolic addition subscale (23 children, 32.9%), compared to symbolic subtraction (18 children, 25.7%) and word problem solving (19 children, 27.1%). Only 8 children (11.4%) consistently used their fingers at least once throughout all three subscales. Of the 32 finger users, 25 still used their fingers at posttest, whereas 7 stopped using their fingers. Of the 38 non-finger users at pretest, a majority of 27 children still did not use their fingers at posttest, while 11 developed into finger users.

All in all, the proportion of finger users increased from 45.7% (32 children) at pretest to 51.4% (36 children) at post-test, corresponding to an increase of 5.7% points. In the intervention group, the proportion of finger users increased by 6.1% points from 57.6% (19 children) to 63.6% (21 children), while in the control group it increased by 5.4% points from 35.1% (13 children) at pretest to 40.5% (15 children) at posttest. Thus, finger use for basic arithmetic developed comparably, irrespective of whether children received or did not receive a finger-based intervention. Inspired by an anonymous reviewer’s comment, we further evaluated whether children in the intervention group might have used their fingers more frequently as a result of the intervention, even though the number of finger users did not increase significantly. Complete data on finger use for all items of the three subscales on basic arithmetic (level III) were available for *n* = 64 at pretest and *n* = 63 children at posttest (i.e., 31 at t1 and 30 at t2 from the intervention and 33 from the control group). As data was not normally distributed, we conducted a nonparametric Mann-Whitney-U test. Results indicated that already at pretest children of the intervention group used their fingers significantly more often than children of the control group (*Mdn* = 2 and *Mdn* = 0; *U* = 343.50, *p*=.014). At posttest, this pattern was even more pronounced (*Mdn* = 5 and *Mdn* = 0, *U* = 265.50, *p*<.001). However, no significant group differences were found with regard to the development of finger use frequency between t1 and t2 (i.e., frequency of finger use t2 - frequency of finger use t1; *Mdn* = 0 and *Mdn* = 0, *U* = 447.00, *p*=.486). To further examine the development of finger use as a consequence of the intervention, we ran the same analysis again including only the group of non-finger users at pretest. Descriptively, finger use increased in this group from t1 to t2 by 0.4 items in the control group and by 2.6 items in the intervention group. However, this group difference was not statistically significant (*Mdn* = 0 and *Mdn* = 0, *U* = 109.00, *p*=.285). In sum, these results indicate that only a few children transitioned from non-finger users to finger users as a result of the intervention (*n* = 5). However, those who did the transition, used their fingers at least descriptively more frequently than comparable children of the control group.

### Characteristics of finger users

To examine the extent to which finger users and non-finger users – irrespective of group assignment – differ in their early numeracy skills, we conducted a detailed analysis comparing these two groups across all three levels of early numeracy skills both at pre- and posttest.

**Table 5:** Descriptives for the groups of finger users and non-finger users at pre- and posttest.

		Finger user (t1)				Non-finger user (t1)			
	Max	*N*	M	SD		*N*	M	SD	Cohen‘s d
**Early numeracy skills (t1)**	74	32	48.56	10.02		38	40.55	12.30	0.71**
Level I: Counting (t1)	32	32	24.09	6.61		38	20.32	7.90	0.52*
Level II: Cardinality (t1)	20	32	16.88	2.25		38	15.74	3.34	0.39
Level III: Basic arithmetic (t1)	22	32	7.59	4.36		38	4.50	3.02	0.84**
		**Finger user (t2)**				**Non-finger user (t2)**			
	**Max**	***N***	***M***	***SD***		***N***	***M***	***SD***	**Cohen‘s** ***d***
**Early numeracy skills (t2)**	74	36	51.89	9.20		34	44.18	13.52	0.67**
Level I: Counting (t2)	32	36	25.42	5.76		34	21.82	7.78	0.53*
Level II: Cardinality (t2)	20	36	17.75	2.35		34	16.24	3.29	0.53*
Level III: Basic arithmetic (t2)	22	36	8.72	4.27		34	5.71	4.51	0.69**

Note. Maximum score points for each variable (Max). ^**^p<.01, ^*^p<.05.

As depicted in Table 5, the group of finger users outperformed the group of non-finger users descriptively with respect to the overall score *early numeracy* and at all levels of early numeracy skills. To further evaluate this, we conducted independent sample *t*-tests comparing finger-user (t1) vs. non-finger users (t1).

Results indicated that finger users at pretest outperformed non-finger users not only on the overall score of early numeracy (t1), *t*(68) = 2.95, *p=*.004, but also on level I, *t*(68) = 2.15, *p=*.035, and level III, *t*(53.7) = 3.39, *p=*.001, while no significant differences were observed for level II, *t*(68) = 1.64, *p=*.106. A similar pattern was observed for the comparison of finger users vs. non-finger users at posttest. Again, finger users outperformed non-finger users on the overall early numeracy score (t2), *t*(57.7) = 2.77, *p*=.007, as well as on level I, *t*(60.7) = 2.19, *p*=.031, level II, *t*(59.4) = 2.20, *p*=.031, and level III, *t*(68) = 2.88, *p=*.005.

For children, who developed from non-finger users to finger users at posttest (*n* = 11 finger use starters) compared to those who stayed non-finger users (*n* = 27 persistent non-finger users), a significant difference in the overall score early numeracy (t2) was found, *t*(36) = 2.38, *p=*.024, *d*=0.70, indicating that finger use starters outperformed persistent non-finger users with respect to early numeracy (t2). Interestingly, this group comparison did not turn significant for pretest early numeracy, *t*(36) = 1.57, *p=*.125, although there was still a medium sized group difference on the descriptive level (*d*=0.56). Thus, finger use starters were already at t1 (descriptively) better with respect to their early numeracy and even extended their lead at t2 compared to persistent non-finger users.

## Discussion

The present study aimed to develop and evaluate a finger-based early numeracy intervention specifically designed to systematically follow early numeracy development according to the model by Krajewski & Schneider^8^ without considering domain-general aspects such as fine motor and finger sensory skills in the program explicitly. To pursue this aim, we developed 12 intervention sessions (30 min each) targeted at enhancing children’s number and counting skills (level I), cardinality understanding and ordering (level II) and basic arithmetic skills (level III) through finger counting, finger number gesturing, and structure-based finger arithmetic. Subsequently, we conducted a controlled intervention study employing a pre-post-test design to evaluate the efficacy of the intervention compared to a business-as-usual control group.

### Efficacy and specificity of the intervention

Results indicated that children of the intervention group significantly outperformed children of the control group at posttest with respect to overall early numeracy while adjusting for pretest performance and fluid reasoning. Yet, it needs to be considered that the effect became marginal (*p*=.054) when spatial working memory was added as a further covariate. In contrast, no significant intervention effects were observed for spatial working memory and fluid reasoning, suggesting that the intervention was not only overall effective but also specific to early numeracy. This pattern of results is also interesting from the perspective of a control analysis. Since the intervention group outperformed the control group in all performance domains at pretest, one could argue that the observed intervention effects represent a Matthew effect (i.e., higher-performing children improve more). Although we cannot completely rule out the influence of pretest differences on the intervention effect besides statistical adjustment in the ANCOVAs; the fact that the intervention group showed an improvement only in early numeracy, but not in fluid reasoning or spatial working memory provides strong evidence for a specific intervention effect despite existing pretest differences.

According to Cohen^48^, the observed effect was medium in size (*η*_*p*_^*2*^ = 0.07). This is in line with effects reported for previous finger-based intervention studies focusing specifically on early numeracy skills without considering domain-general abilities such as fine motor or finger sensory skills in the intervention^18,20,23^. By contrast, intervention studies that also included non-mathematical fine motor and finger sensory tasks into the intervention, reported smaller effect sizes^17,21^ or even no significant intervention effects^22^. This pattern is further substantiated when intervention time is taken into account. The time spent on the present intervention was rather low with 12 sessions of 30 min (see Jay & Betenson^18^ for similar intervention time) and even lower for studies focusing on specific aspects of early numeracy skills (e.g., addition in Poletti et al.^20^). However, interventions including also non-mathematical fine motor and finger sensory skills, invested comparably more time while receiving less pronounced improvements^17,21,22^. Even though all of these studies are of course only partially comparable in terms of intervention content and measures taken, the present findings suggest that finger-based early numeracy interventions are effective even when fine motor and finger sensory skills are not considered explicitly in intervention activities (see also Lewis and Weixler^25^. In fact, it might be that finger use has to be intentionally linked with numbers in order to have beneficial effects. However, the present study is not conclusive on this aspect. The fact that no activities were included that specifically targeted domain-general skills such as fine motor abilities or finger sensory skills does of course not necessarily mean that these skills did not play a role.

Considering specific levels of early numeracy skills, the present study indicated a medium sized, significant intervention effect for level I numeracy skills (numbers and counting), while no significant effect was observed for level II (cardinality understanding and ordering) and the effect was only marginally significant for level III (basic arithmetic). This suggests that children made significant progress with respect to verbal counting, naming successor and predecessor numbers, and digit knowledge (level I) due to the finger-based intervention. As these were (amongst others) major topics of the intervention, these results seem reasonable and in line with our expectations.

Interestingly and contrary to our hypothesis, we did not observe a significant intervention effect for cardinality understanding and ordering (level II). A possible explanation for this might be that – besides excluding children scoring at ≥ 85% on overall early numeracy skills at pretest – there still were indications of a ceiling effect at pretest specifically for level II (81% accuracy on average). By contrast, for level I and level III, overall pretest performance was in the medium range with 69% and 27% of tasks solved correctly. However, an additional analysis excluding children scoring both ≥ 85% on overall early numeracy and ≥ 85% on cardinality understanding and ordering (level II) significantly reduced the overall sample size (*n* = 33) and suggested that pretest differences alone cannot account for the null results. Interestingly, children in our sample also performed better on Level II than on Level I, which actually argues against a hierarchical progression between the two levels. Indeed, cardinality understanding might be particularly challenging to measure, as substantiated by recent results reported by Thevenot and Krenger^15^. Thus, future studies should carefully think about how to measure potential intervention effects on cardinality understanding in 5- to 6-year-olds.

As regards level III (basic arithmetic), intervention effects were small and no longer significant when spatial working memory was included as control variable. A possible explanation for this pattern of results might be that most sessions on basic arithmetic (sessions 8 to 12) focused primarily on number completion (i.e., completing numbers up to 5 and 10 or the “how-many-more” task). These tasks rely on a different finger-based strategy (i.e., representing the given quantity with fingers and identifying how many are missing to 5/10 etc.) compared to solving additions or subtractions with fingers (e.g., representing two quantities and combining them), which would have been needed to solve the symbolic addition and subtraction tasks during posttest.

The small intervention effects for basic arithmetic (level III) go along with the observation that the number of finger users in arithmetic did not particularly increase as a result of the finger-based intervention. During the intervention it became obvious that some children continuously needed encouragement to use their fingers to help model a task. For pre- and posttest, children were neither encouraged nor hindered to use their fingers to solve the arithmetic tasks which may have led children, who were still unsure, to simply not choosing to use them. This explanation is supported by further exploratory analyses within the group of non-finger users only. These revealed that, although only a few children of the intervention group transitioned from non-finger users to finger users, those who made the transition used their fingers more frequently, at least descriptively, than comparable children of the control group. All in all, there is much to suggest that it was mainly not enough time to fully learn the different arithmetic finger strategies and apply them independently. In future studies, it would be reasonable to further enrich the intervention and specifically include – besides sessions on number completion to 5 and 10 - more sessions targeted at finger-based addition strategies.

One limitation of the present study concerns the exclusion of children who performed at ceiling (≥ 85%). While this criterion is justified as no further, at least measurable performance increase was to be expected from these children, this non-random exclusion reduced statistical power and may have affected the representativity of the sample. Furthermore, as the finger-training group was compared to a business-as-usual control group receiving less numeracy instruction during the intervention period, we cannot fully rule out that some of the observed effects were influenced by the additional practice opportunities provided through the intervention sessions rather than by the specific content of the intervention itself.

#### Development and characteristics of finger use

To examine characteristics of finger users vs. non-finger users we applied the procedure reported by Krenger and Thevenot^15^. Based on observations during the basic arithmetic tasks, we created groups of finger-users (i.e., finger use observed at least once) and non-finger users (i.e., no finger use observed) both at pretest as well as at posttest. In our sample of 5- to 6-year-olds, 46% of children used their fingers to solve at least one of the given symbolic addition, subtraction or word problems at pretest. This number even increased at posttest with 51% of children using their fingers. Interestingly, this increase was not only observed for the intervention group, but also for the control group, suggesting a development rather than a specific intervention effect in this case. In this context, it should be noted that for the word problem subscale - but not for the symbolic addition and subtraction subscale - manipulatives were available to the children. As children could solve the tasks with the manipulatives instead, fingers were used less frequently during this task. However, this appears to have had little impact on the overall number of finger users.

The number of observed finger users is comparable to what was reported by Björklund et al.^13^, who observed 44% of their 4- to 5-year-olds to solve a subtraction word problem using fingers. Only 38% of children (age 5 to 6 years) were reported to use their fingers in the study by Krenger and Thevenot^15^, which might be due to the fact, that finger use was examined while solving rather easy addition problems compared to the broader range of task types which built the basis for the classification of finger users in the present study. Overall, the present results substantiate previous findings suggesting that about a third to about half of children seem to use their finger to solve arithmetic tasks spontaneously between age 4 and 6. Moreover, the use of fingers does not appear to be merely a developmental phenomenon, whose frequency depends solely on the age group studied. Varying frequencies observed in previous studies may also indicate that the actual type of task may influence whether or not fingers are used. However, finger use has not been systematically compared so far across different types of tasks, which could provide further insights into the phenomenon.

A closer look at the characteristics of finger users vs. non-finger users further revealed that finger users outperformed non-finger users both at pre- and posttest with respect to early numeracy. Interestingly, differences were not only evident at level III (basic arithmetic) but also at level I (numbers and counting) and, at least at posttest, also at level II (cardinality understanding and ordering). In all cases, finger users outperformed non-finger users. This means that finger users were not only better at arithmetic, which seems reasonable because fingers provide a visualization of the abstract concepts of addition and subtraction^14,15,49^. They also outperformed non-finger users with respect to cardinality understanding and counting. The latter was also reported by Krenger and Thevenot^15^, who found that at least at age 4 to 5, finger users significantly outperformed non-finger users with respect to object counting.

## Conclusion

In summary, the present study indicated that a finger-based early numeracy intervention that is systematically aligned with models of early numeracy development can effectively promote basic numeracy skills in 5- to 6-year-olds, even though domain-general abilities such as fine motor or finger sensory skills were not covered in the intervention. The intervention revealed a medium-sized, significant effect on overall early numeracy performance (marginally significant when spatial working memory was added as additional covariate). The intervention effect was particularly driven by number knowledge and counting (level I). Effects on basic arithmetic (level III) were found to be marginally significant, while no significant effect was observed for cardinality understanding and ordering (level II). Furthermore, an analysis of the development and characteristics of finger users versus non-finger users highlighted that the number of finger user for basic arithmetic developed comparably and irrespective of whether children received or did not receive a finger-based intervention. Although only a few children of the intervention group transitioned from non-finger users to finger users, those who made the transition used their fingers more frequently than comparable children of the control group, but only on a descriptive level. In general, those children who employed finger-based strategies to calculate, systematically outperformed their peers in early numeracy. Taken together, these results suggest that finger use in this area is a powerful and beneficial approach to promoting early numeracy.

## Supplementary Information

Below is the link to the electronic supplementary material.

Supplementary Material 1

Supplementary Material 2

## Data Availability

Data will be made available on request by the corresponding author of this paper.
