# Supplementary material for: Design and evaluation of a systematic finger-based intervention for early numeracy in 5- to 6-year-olds
Source: Sci Rep. 2026 Mar 26;16:10495. doi: 10.1038/s41598-026-43286-1 (PMC13031659; doi:10.1038/s41598-026-43286-1)
Supplement: Supplementary file 1 — Supplementary Material 1 [file 41598_2026_43286_MOESM1_ESM.pdf]

Supplementary online material: Table 1

Descriptives of early numeracy level I, II, and III as well as for all subscales

		Control group			Intervention group			
	Max	N	M	SD	N	M	SD	Cohen‘s d
**Level I: Counting (t1)**	32	37	21.38	8.24	33	22.79	6.69	.19
Number sequence (t1)	12	37	8.22	2.90	33	8.15	3.03	-.02
Digit knowledge (t1)	10	37	13.16	5.83	33	14.64	4.49	.28
**Level II: Cardinality (t1**	20	37	16.11	2.69	33	16.42	3.22	.11
Number concept (t1)	5	37	4.24	1.19	33	4.52	1.09	.24
Number seriation (t1)	7	37	5.59	1.19	33	5.48	1.37	-.09
Number comparison (t1)	8	37	6.27	1.50	33	6.42	1.75	.09
**Level III: Basic arithmetic (t1)**	22	37	5.38	4.03	33	6.52	3.90	.29
Word problems (t1)	6	37	2.68	1.65	33	2.94	1.41	.17
Symbolic addition (t1)	10	37	2.11	2.49	33	3.00	2.59	.35
Symbolic subtraction (t1)	6	37	0.59	1.24	33	0.58	1.35	-.01
**Level I: Counting (t2)**	32	37	22.32	7.53	33	25.18	6.13	.41
Number sequence (t2)	12	37	8.57	2.72	33	9.39	2.47	.32
Digit knowledge (t2)	10	37	13.76	5.37	33	15.79	4.58	.41
**Level II: Cardinality (t2)**	20	37	16.78	2.81	33	17.27	3.07	.17
Number concept (t2)	5	37	4.59	0.96	33	4.76	0.50	.21
Number seriation (t2)	7	37	5.97	1.36	33	5.67	1.76	-.20
Number comparison (t2)	8	37	6.22	1.75	33	6.85	1.68	.37
**Level III: Basic arithmetic (t2)**	22	37	6.51	4.48	33	8.52	4.75	.43
Word problems (t2)	6	37	3.03	1.42	33	3.30	1.49	.19
Symbolic addition (t2)	10	37	2.89	2.94	33	4.48	3.03	.53
Symbolic subtraction (t2)	6	37	0.59	1.26	33	0.73	1.42	.10
