# Supplementary material for: Design and evaluation of a systematic finger-based intervention for early numeracy in 5- to 6-year-olds
Source: Sci Rep. 2026 Mar 26;16:10495. doi: 10.1038/s41598-026-43286-1 (PMC13031659; doi:10.1038/s41598-026-43286-1)
Supplement: Supplementary file 2 — Supplementary Material 2 [file 41598_2026_43286_MOESM2_ESM.pdf]

Supplementary material:

Detailed instruction for intervention session 4 (Numbers 6 and 7) and session 9 (Number relations – 10) translated from German

4

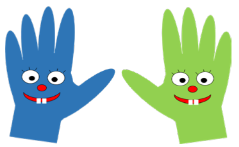

Numbers 6 and 7

Key questions: What do the numbers 6 and 7 look like? In what order can the numbers from 0 to 7 be arranged? What finger pattern emerges when counting with fingers up to 6 or 7, and how is this pattern structured?

Insights:
1. When counting with fingers, finger number gestures are formed that always look the same if one begins counting with the thumb.
2. Finger number gestures can be formed sequentially (i.e., extending fingers one after another) or simultaneously (i.e., extending fingers all at once).
3. Each finger number gesture consists of extended and folded fingers.
4. Numbers can be ordered according to the counting sequence.

Material: Puppets “Ed & Ted”, digit cards and all finger pattern cards from 0 to 7, finger-number booklet, pencil, colored pencils/crayons

Step 1: Numbers and finger number gestures - Ed and Ted form finger number gestures sequentially or simultaneously

Ed and Ted (i.e., the teacher wearing the two finger gloves Ed and Ted) ask the children whether they remember which numbers in the finger-number booklet have already been worked on. The children name the numbers, and Ed and Ted place the digit cards from 0 to 5 on the table accordingly. Ed then chooses the digit card 3 and wants to form the corresponding finger number gesture. He thinks for a moment and forms the finger number gesture sequentially (i.e., finger counting to 3). Ted responds by saying that he already remembers that in the finger number gesture for the number 3, the thumb, index finger, and middle finger are extended, while the other two fingers remain folded. He forms the finger number gesture all at once (i.e., simultaneously). The children realize that finger number gestures can be represented either sequentially or simultaneously if one already knows which fingers need to be extended!

Now, each child may in turn choose one of the remaining digit cards and form the corresponding finger number gesture - either sequentially by counting up with fingers or if already possible simultaneously. Next, Ed and Ted show the digit card 6 to children. What number is this? Together with Ed and Ted, they now count up to this number (IMPORTANT: when counting – palm facing up; when representing the quantity – turn the palm down onto the table!). Then, the structure of the finger pattern for the number 6 is discussed in more detail: How many fingers are extended on one hand? How many are folded? And how many fingers are extended or folded on the other hand? The digit card 6 is also handed to a child. The same procedure is then followed with the digit card 7.

University of Tuebingen | Hector Research Institute for education sciences and psychology | Dr. Stephanie Rösch | stephanie.roesch@uni-tuebingen.de

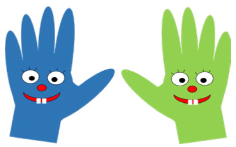

Step 2: Eagle-eye game

Each child has one or two digit cards in front of them from step 1. In the Eagle-Eye Game, the children imagine they are eagles hunting for “mice.” The mice are the finger pattern cards from 0 to 7. The teacher places a finger pattern card in the middle of the table. The child, who has the matching digit card, quickly snatches the finger pattern card and says the number aloud. Meanwhile, the “mouse” tries to escape (i.e., the teacher moves the finger pattern card on the table). Once the child has caught the finger pattern card, the teacher puts the next card on the table. The game continues until no finger pattern cards remain. Finally, the digit and finger pattern cards are jointly arranged on the table from in ascending left-to-right order (top row: digit cards; bottom row: corresponding finger pattern cards).

Step 3: The finger-number booklet– Numbers 6 and 7

The numbers and finger number gestures for 6 and 7 are now worked on in the finger-number booklet. All children open the page for the number 6. What is special about the number 6? What is special about the number 7? Each child traces the line with their finger (i.e., depicted as a road with the finger representing a car driving down the road). Then, the line is traced three times with a crayon, allowing the structure of the number to be experienced not only visually but also motorically. Next, each child forms the finger number gesture for 6 on a blank page of the finger number booklet and the teacher traces the outline of the child’s hand. The child then traces the outline of the finger pattern with a colored pencil. The same process is then repeated with the number 7.

Step 4: Blindfold game with numbers and finger number gestures

Finally, the traced numbers are identified with closed eyes. The activity is done in pairs. One child begins and closes their eyes. The other child guides their partner’s index finger along the tracing of a number in the finger-number booklet and then closes the book. *Which number is it?* The child names the number and then forms the respective finger number gesture - either sequentially or if already possible simultaneously.

University of Tuebingen | Hector Research Institute for education sciences and psychology | Dr. Stephanie Rösch | stephanie.roesch@uni-tuebingen.de

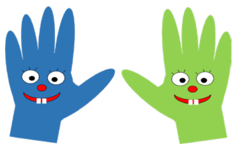

Number relations (10)

Key questions: How many are missing to reach the number 10, and how can this be determined using fingers?

Insights:
1. Numbers/quantities are related.
2. The number missing up to 10 can be determined by folded and extended fingers.

Material: Puppets “Ed & Ted”, large number caterpillar, green leaf for covering, number dominoes, treasure chest with 10 glass stones, score sheet, number dice 0–9.

Step 1: Ed and Ted and the large number caterpillar

The teacher asks the children if they remember what Ed and Ted found on their way to kindergarten last time. Right, a small number caterpillar with 5 beads. Today, they have found another caterpillar, but a much longer one! Ed and Ted show the large number caterpillar to the children, and together they notice that it consists of 5 green and 5 blue beads. That fits perfectly because the caterpillar has 5 green beads, and we have 5 fingers on one hand. And it has 5 blue beads, and we have another 5 fingers on the other hand! This caterpillar is also shy and likes to hide. The teacher hides the caterpillar under a leaf, letting 7 beads show and asking the children: “How many beads can you see?” Right, 7 beads. “Show me 7 fingers and try to figure out how many beads are hidden!” The children realize that by looking at the folded fingers, they can see how many beads of the caterpillar are hidden. 3 fingers are folded. So there are 3 beads hidden under the leaf. In this way, more puzzles are solved. Subsequently, the children may hide the number caterpillar themselves and create a puzzle for their friends.

University of Tuebingen | Hector Research Institute for education sciences and psychology | Dr. Stephanie Rösch | stephanie.roesch@uni-tuebingen.de

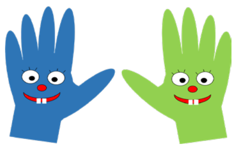

Step 2: Number domino

The teacher explains to the children that Ed and Ted have brought a number domino for them. All cards are laid out on the table and the teacher explains that the number domino starts with the starting card, and it is the children's task to place all the cards in order along a path to the target card. The rule is that each card added must combine with the number on the previous card to make 10. The domino is now played together. Each child takes turns placing a card and, with the help of their fingers, determines which number needs to be added.

Step 3: Ed and Ted's treasure chest

The teacher asks the children if they remember what Ed's and Ted's treasures are. Right, glass stones! The teacher shows a small treasure chest with glass stones inside. The children first count how many stones are in it. Correct, there are 10 stones! That works perfectly, because we also have exactly 10 fingers. The teacher explains that Ed and Ted now want to play a game with them. In this game, all the children play together against Ed and Ted. In each round, the goal is to take as many of the 10 stones from Ed and Ted's treasure chest as possible. If the children take more beads than are left in the chest, they get a point. If more beads remain in the chest, Ed and Ted get a point. The first to reach 6 points wins. How many stones the children get in a round is determined by rolling the number dice (0–9). If a 0 is rolled, the children get to choose how many stones they want. The first child begins and rolls a 6, for example. The educator secretly takes 6 stones from the treasure chest and asks the child: "You have 6 stones. How many stones are still in the treasure chest?" The teacher encourages the child to solve this using their fingers (i.e., form 6 with their fingers and use the folded fingers to figure out how many are missing to reach 10). Correct, there are 4 stones left in the chest! Then the educator asks: "You have 6 stones. Ed and Ted have 4 stones. Who has more stones now?" Right, the children have more glass stones and therefore earn a point. They record this on the score sheet. Now it's the next child's turn, who rolls the dice and uses their fingers to determine how many beads are left in the treasure chest, awarding a point either to the children or to Ed and Ted.
